# Is Idiopathic Granulomatous Mastitis a Subgroup of Systemic Lupus Erythematosus? A Preliminary Study

**DOI:** 10.3390/jcm13206242

**Published:** 2024-10-19

**Authors:** Murat Toprak, Nursen Toprak

**Affiliations:** 1 Department of Physical Medicine and Rehabilitation, Medical Faculty, Van Yüzüncü Yıl University, Van 65090, Turkey; 2 Department of Radiology, Medical Faculty, Van Yüzüncü Yıl University, Van 65090, Turkey; nursentoprak@yyu.edu.tr

**Keywords:** idiopathic granulomatous mastitis, systemic lupus erythematosus, systemic lupus erythematosus risk profile index, autoimmunity, autoantibodies

## Abstract

**Objective:** The study aimed to use the systemic lupus erythematosus risk probability index (SLERPI) to assess if patients with idiopathic granulomatous mastitis (IGM) meet the criteria for systemic lupus erythematosus (SLE). **Methods:** A total of 62 patients with IGM and 55 age- and sex-matched healthy controls (HC) were enrolled. The study included patients who were over 18 years old and had been diagnosed with IGM using a true-cut biopsy. The participants’ demographic, clinical, and laboratory data were recorded in detail. The presence of autoantibodies, such as RF, CCP, C3, C4, ANA, ENA profile, and Anti-dsDNA was documented. For the detection of SLE in IGM patients, we used the SLERPI (SLE risk probability index). **Results:** A total of 62 patients diagnosed with idiopathic granulomatous mastitis (age 35.22 ± 8.34, BMI 27.15 ± 3.41) were compared to 55 healthy controls (age 32.54 ± 8.67, BMI 26.97 ± 3.54). The present study assessed the performance of SLERPI in IGM, and SLERPI positivity was observed in 12 out of 62 (19.4%) IGM patients. There was a significant difference in arthritis and ANA levels in the SLERPI subgroups (*p* < 001). **Conclusions:** The SLERPI index can be utilized to identify patients suspected of having systemic lupus erythematosus (SLE) in the IGM cohort.

## 1. Introduction

Idiopathic granulomatous mastitis (IGM) is a rare chronic autoimmune disease. It is characterized by granulomas [1,2]. Notably, young women may be susceptible to developing a painful breast mass. The condition primarily affects women between the ages of 20 and 40 [3]. It is hypothesized that IGM has an autoinflammatory origin; however, the precise etiology remains unknown [4]. A definitive diagnosis is based on thorough histologic evaluation of non-caseating granulomatous inflammation in a true-cut biopsy [5]. The most commonly employed therapeutic modalities for immune suppression encompass corticosteroid (CS), methotrexate (MTX), azathioprine (AZA), and surgical procedures such as mastectomy or local excision [6].

Systemic lupus erythematosus (SLE) is an autoimmune disease characterized by the aberrant recognition of self-antigens [7]. The clinical manifestations of SLE encompass a range of symptoms, including cutaneous rashes, arthritis, pleurisy, alopecia, and lupus nephritis [8]. There is a greater prevalence of SLE in women of reproductive age than in men [9]. The diagnosis of autoimmune disease necessitates the presence of specific autoantibodies: ANA, anti-dsDNA, anti-Ro, anti-La, antiphospholipid, ENA, C3, and C4. The European League Against Rheumatism (EULAR) and the American College of Rheumatology (ACR) have developed a set of criteria, known as the 2019 EULAR/ACR criteria, which are widely used for the diagnosis of lupus [10]. The Systemic Lupus International Collaborating Clinics (SLICC) have also established a set of criteria, known as the SLICC criteria, which are similarly used for the diagnosis of lupus [11]. The management of SLE flares involves the utilization of glucocorticoids and/or immunosuppressants.

SLE is a heterogeneous group of diseases that share a common clinical feature of systemic autoimmunity [12]. SLE has various subtypes including drug-induced lupus erythematosus (DILE), cutaneous lupus erythematosus (CLE), which consists of discoid lupus erythematosus (DLE), and subacute cutaneous lupus erythematosus (SCLE), juvenile lupus erythematosus, and neonatal lupus erythematosus [13]. Some authors use the terms “incomplete lupus”, “early lupus”, “possible lupus”, and “latent lupus” to describe patients with SLE-like features who do not meet the established classification criteria [14].

Both of these autoimmune diseases are characterized by a relapsing–remitting course of progression and primarily affect young women [1,3,5]. Both diseases are associated with birth and/or breastfeeding [1,3,9]. Arthritis/arthralgia, erythema nodosum, acute phase reactant, and ANA positivity are commonly observed in patients with this condition [1,5,8,9]. Furthermore, immunosuppressive drugs are commonly employed in medical treatment [1,5,6,7,8,9]. Given their shared characteristics, is it possible for IGM to be considered a subtype of SLE?

The appearance of SLE is highly variable, making diagnosis challenging. In recent years, indices like the systemic lupus erythematosus risk probability index (SLERPI) have been used for early disease diagnosis and classification. Adamichou et al. [15] developed the SLERPI for early diagnosis and treatment of SLE. SLERPI is an index that predicts SLE using 14 clinical and serological features. A case is classified as SLE if the score is greater than seven, and non-SLE if the score is less than or equal to 7. The index demonstrates high sensitivity (95.1%) and specificity (93.7%) for the early stages of the disease. It is currently unclear whether the index predicts SLE development in IGM patients.

The study aimed to determine if IGM qualifies as a subset of SLE based on SLERPI criteria.

## 2. Materials and Methods

### 2.1. Ethics Statement

Approval for our study protocol (Approval number: B.30.2.YYU.0.01.00.00/68) was obtained from the University Medical Research Ethics Committee. The study protocol was developed in accordance with the ethical standards set forth in the Helsinki Declaration. The study protocol was elucidated in exhaustive detail, and written consent was obtained from all participants.

### 2.2. Patients

A total of 62 patients with IGM and 55 age- and sex-matched healthy controls (HC) were recruited from the University Medical Faculty Hospital between March 2023 and August 2023 for inclusion in this prospective study.

### 2.3. Clinical Parameters

During this study, the demographic and clinical data of the participants were recorded. This included information on age, gender, occupation, body weight (kg), height (cm), body mass index (kg/m^2^), smoking habits, comorbidities, presence of IGM disease, disease duration, treatments received, arthritis/arthralgia, morning stiffness, and patients’ pain assessment visual analogue scales (VAS), such as patients’ global VAS, physician VAS, and patients’ VAS. A comprehensive panel of blood tests was conducted, encompassing a complete blood count, biochemistry, serum creatinine, urine analysis, estimated glomerular filtration rate, proteinuria (>500 mg/24 h), and complement 3 and 4. Additionally, the presence of the rheumatoid factor (RF), anti-citrullinated protein antibodies (anti-CCP), C-reactive protein (CRP), and the erythrocyte sedimentation rate (ESR) was documented in both IGM patients and healthy controls. The following tests were conducted: ANA, anti-dsDNA, and ENA. The reference values for RF and anti-CCP were <15 IU/mL and <5 U/mL, respectively. The term ANA positivity indicates HEp-2 IFA positivity with a titer of 1:80 or higher. The panel of antigens included in the ENA test was as follows: Smith (Sm), RNP, Scl70, anti-Mi-2, Jo-1, CENP-B, ribosomal P, histone, PM-Scl-100, Ro, La, and PCNA.

### 2.4. Inclusion and Exclusion Criteria

The inclusion criteria for the IGM group were as follows: The patient cohort comprised individuals aged 18 years and over who had been diagnosed with IGM by means of a true-cut biopsy. The control group consisted of individuals aged 18 years and older, without any autoimmune or chronic diseases. Patients who did not provide written consent or whose records were incomplete were excluded from the study.

### 2.5. Assessing the Symptoms of Systemic Lupus Erythematosus

In order to identify SLE symptoms in IGM patients, we utilized the SLE risk probability index (SLERPI) proposed by Adamichou et al. (Table 1) [15]. Once the threshold was set above 7 (out of a maximum score of 30.5), the sensitivity, specificity, and accuracy were estimated at 94.2%, 94.4%, and 94.2%, respectively.

### 2.6. Statistical Analysis

Descriptive statistics for the continuous variables were presented as mean, standard deviation, minimum, and maximum values while count and percentages were presented for categorical variables. Normality assumption of the continuous variables was tested with the Kolmogov–Simirnov test. After the normality test, an independent *t*-test (Student’s *t*-test) was used to compare group means. For determining linear relationships among the variables, Pearson correlation analysis was carried out. The statistical analysis was conducted using the Statistical Package for the Social Sciences (SPSS) software, version 23.0, produced by IBM. The level of significance was set at 5%.

## 3. Results

A total of 62 cases (age 35.22 ± 8.34, BMI 27.15 ± 3.41) were diagnosed with IGM, and 55 healthy controls (age 32.54 ± 8.67, BMI 26.97 ± 3.54) were included in the study (Table 2). The IGM patient group and the healthy control group consisted only of female subjects.

Significant variations were noted in the RBC count, hemoglobin, ESR, and ANA levels among the groups (*p* < 0.001). Table 3 presents a comparison of the biochemical and immunological characteristics of patients with IGM and HC.

**Figure 1:**
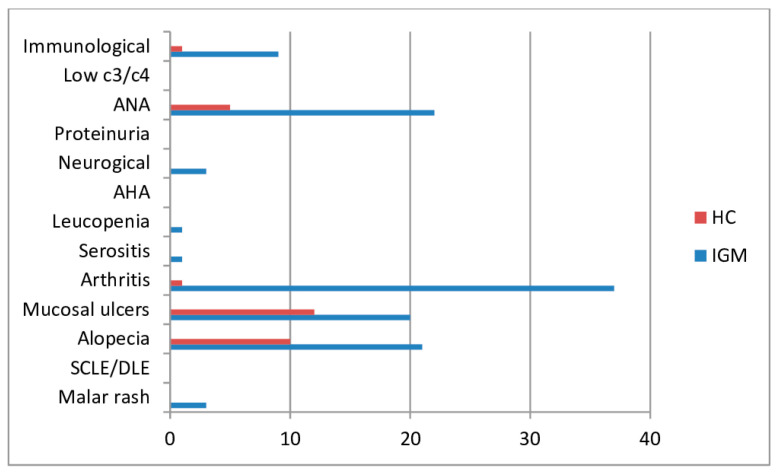
Graphical representation of the findings between the IGM and HC groups in SLERPI.

A significant difference was found in the prevalence of arthritis and ANA among the SLERPI (positive/negative) (*p* < 0.001), while no such distinction was observed among the other parameters (Table 4, Figure 1). SLERPI was ≥7 in 12 of 62 patients. A significant difference was observed in the levels of arthritis and ANA between SLERPI (positive/negative) IGM patients (*p* < 0.001). However, no distinction was evident in the other parameters (Table 5).

## 4. Discussion

While there have been some cases evaluating the coexistence of IGM and SLE, to the best of our knowledge, this is the first study to evaluate SLE in patients with IGM. In this study, patients with IGM were assessed using the SLERPI, which yielded positive results in one out of five patients. The disease mainly affects women of reproductive age. Additionally, we observed increased levels of arthralgia and ANA.

The heterogeneous nature of SLE has sparked debate on whether it is a single disease with varied phenotypes or a combination of different diseases with diverse pathogenic mechanisms. It is expected that as our knowledge of this subject advances, additional subcategories of SLE disease will become apparent [16].

The evidence suggests that SLE symptoms may be more prevalent among individuals with IGM; however, the data are not conclusive. A number of studies, case reports, and case series have been published that support the relationship between IGM and SLE [1,5,17,18]. Although the SLICC and EULAR/ACR criteria are frequently employed for the classification of SLE, there is a growing tendency to utilize practical classification criteria such as SLERPI for the early diagnosis of the disease. The SLERPI index, designed to assist in the diagnosis of SLE, was used to evaluate a total of 62 IGM patients.

IGM and SLE share several similarities. Evidence in the available literature suggests that both diseases occur in young women, specifically at their childbirth and breastfeeding age, and that they respond well to steroid and immunosuppressive treatments [1,3,5,6,7,8,9]. Furthermore, recurrences and extra-breast involvement, such as erythema nodosum, arthritis, and alopecia, have been observed [5,6]. Autoantibody positivity, as indicated by the presence of ANA [1,5], T-lymphocyte dominance in immunohistochemical studies [6], and the presence of HLA-DR*17 [19] antigens, is a common feature of both SLE and IGM.

The mean age at diagnosis in our study was 35.22 ± 8.34 years, which is similar to the age reported by Abbi et al. (35.8 ± 9.4) [20]. A review of 3060 IGM cases showed a median age of 36 years (range: 19–49 years) [21,22]. Both groups consisted solely of female patients. In our study, 37 out of 62 patients with IGM (59.7%) showed arthritis, while four (6.5%) had erythema nodosum. A systematic review of 3060 cases indicated that 8% showed erythema nodosum, consistent with the previous study [21].

It has been observed that certain autoimmune tests, such as ANA, RF, CCP, and ENA, may produce different results in patients with IGM. Asoglu et al. [23] found that all 18 patients in their series had negative RF levels. However, in Ozel et al.’s [24] study of eight IGM patients, six tested positive for RF. Koksal et al. [25] found no statistically significant difference in the prevalence of RF among the IGMA (IGM active disease) group (10%), the IGMR (IGM remission) group (16.1%), and the control group (13.3%). Two out of the patients (6.7%) in the IGMA group tested positive for anti-CCP antibodies. In the course of our investigation, we noticed that RF was present at a low titer in three patients. To ensure accuracy, we conducted repeated RF tests for control purposes and found that all CCP tests were negative. The study’s relatively limited sample size likely impacted the number of recorded cases, and increasing the sample size could lead to more favorable outcomes.

Antinuclear antibodies (ANA) are used as diagnostic markers for various systemic autoimmune disorders, such as SLE, systemic sclerosis (SSc), Sjogren’s syndrome (SS), and mixed connective tissue disease (MCTD). It is estimated that between 10 and 30% of individuals without clinical symptoms may have low levels of ANA in their serum. The results of our study indicated that 35.5% of the cases tested positive for ANA in the IGM. The test results show that 20 out of 22 patients tested positive with a 1:80 ratio, one patient tested positive with a 1:160 ratio, and one patient tested positive with a 1:320 ratio. In a study conducted by Xu et al. [26], 64.10% (50/78) of patients diagnosed with non-lactational mastitis exhibited the presence of antinuclear antibodies, characterized by granular and cytoplasmic granular fluorescence patterns. In a study conducted by Altintoprak et al. [22] on 26 IGM patients, it was found that 80.8% of the patients exhibited ANA positivity with a titer of 1:40. Additionally, 34.6% exhibited ANA positivity with a titer of 1:100, and 7.8% exhibited moderate ANA positivity with a titer of 1:320. The study findings indicate a high prevalence of ANA positivity among IGM patients. Asoglu et al. [23] reported that all 18 patients in their series had negative results for ANA. Nevertheless, the study conducted by Ozel et al. [24] with eight IGM patients revealed that two patients tested positive for both ANA and anti-dsDNA. In a study by Köksal et al. [25], ANA positivity was found in two out of thirty-one patients (6.5%) in the IGM group.

In a study conducted by Altintoprak et al. [22], it was observed that 11 out of 26 patients (42.3%) showed ENA positivity, especially in response to Ro-52, at a 1:40 titer, while only two out of twenty-six patients (7.8%) demonstrated ENA positivity at a 1:100 titer. The ENA antibody cohort consisted of a small number of patients, each with diverse profiles. The cohort included three patients with nucleosome antibodies, two with PCNA antibodies, one with PM-Scl antibodies, one with SS-B/La antibodies, and one with U1-RNP antibodies. Additionally, one patient had antibodies to both cANCA and another antigen. This study found that the anti-dsDNA antibody was negative, which is consistent with the findings of the study by Koksal et al. [25].

SLE presents with significant variation in its symptoms, which can affect multiple body systems or be confined to a specific organ or region. Despite using classification criteria to identify homogeneous patient groups in clinical trials, these same criteria are often used for diagnosis in clinical practice [27]. The newly proposed classification criteria aim to improve both sensitivity and specificity [28]. An early diagnosis of SLE is of paramount importance. In comparison to the SLICC-2012 and EULAR/ACR-2019 criteria, the SLERPI criteria have a smaller number of items and are more straightforward to apply [27]. In a study conducted by Adamichou and colleagues, they observed an accuracy rate of 94.2% in cases where the SLERPI score > 7 [15]. The objective of our study was to evaluate the performance of SLERPI in IGM patients. Our findings revealed that SLERPI was observed to be positive in 19.4% (12/62) of IGM cases. Patients in the SLERPI of >7 groups had a higher prevalence of cutaneous manifestations, including malar rash, maculopapular rash, alopecia, arthritis, and trace amounts of proteinuria. They also had a higher incidence of ANA positivity. The interstitial lung disease (ILD) feature’s presence made it unlikely that the patient had SLE. No patients in the study cohort exhibited any indications of ILD. A review of the literature revealed no studies in which SLERPI was used in patients with IGM. Erden et al. [29] conducted SLERPI on 422 UCTD patients, identifying 39 cases (9.2%) meeting the SLE criteria.

Several case reports suggest a potential correlation between IGM, EN, and arthritis, indicating the existence of a common underlying pathophysiological mechanism known as the GMENA (granulomatous mastitis, erythema nodosum, and arthritis) syndrome [30,31]. It is our contention that incorporating laboratory markers, such as autoantibodies, into this classification system would facilitate the identification process.

The question thus arises as to whether IGM constitutes a subgroup of SLE or whether it represents a pre-lupus phase in the process leading to SLE. Alternatively, could it be a distinct disease entity, designated as “latent”, “possible”, or “incomplete lupus”, for which the long-term evolution is uncertain? As our understanding of the etiopathogenesis of IGM and SLE deepens, we hope to address the outstanding questions on this subject. Moreover, we posit that the acquisition of long-term follow-up data from IGM patients will facilitate the elucidation of these questions.

It should be noted that the findings of our study are subject to certain limitations. The sample size was relatively modest, which underscores the importance of conducting multicenter studies with a larger sample size. It should be noted that the symptoms (such as alopecia, mucosal ulcers, arthritis, and skin lesions) were self-reported, potentially introducing bias in the results. It is possible that some clinical information may have been overlooked or underestimated. Finally, it is important to note that SLERPI cannot be compared to SLICC and ACR/EULAR criteria. The diagnosis of SLE in patients with IGM will be the subject of a future study in which we will report on SLICC-2012 and ACR/EULAR-2019 criteria.

## 5. Conclusions

One in five patients with IGM exhibited positive results for SLERPI. The classification criteria (SLERPI) provide a valuable tool for the comprehension of SLE symptoms in IGM patients. The utilization of SLERPI allows clinicians to receive earlier alerts. However, it should be noted that this study was preliminary. Further investigation is required to substantiate the efficacy of SLERPI in IGM, particularly with a larger patient cohort. Furthermore, it is important to assess the SLERPI 7 group among IGM patients in accordance with the criteria for SLE.

## Figures and Tables

**Table 1:** The SLE risk probability index.

Feature	Score
Malar rash or maculopapular rash	3
Subacute cutaneous lupus erythematosus or discoid lupus erythematosus	2
Alopecia	1.5
Mucosal ulcers	1
Arthritis	2
Serositis	1.5
Leucopenia < 4000/μL (at least once)	1.5
Thrombocytopenia or autoimmune hemolytic anemia	4.5
Neurological disorder	1.5
Proteinuria > 500 mg/24 h	4.5
ANA	3
Low C3 and C4	2
Immunological disorder (any of anti-DNA, anti-Sm, anti-phospholipid antibodies)	2.5
Interstitial lung disease	–1
SLE if total score > 7	

SLE, systemic lupus erythematosus; ANA, antinuclear antibodies; C3, complement proteins C3; C4, complement proteins C4.

**Table 2:** Baseline demographic and clinical characteristics of patients with IGM and HC.

Variables, n (%)	IGM (n = 62)	HC (n = 55)	*p*-Value
Age, years, mean (SD)	35.22 ±8.34	32.54 ±8.67	0.091
BMI (kg/m^2^)	27.15 ± 3.41	26.97 ± 3.54	0.779
Smoking	15 (24.2)	14 (25.5)	0.075
Disease duration, months, mean (SD)	33.82 ± 29.16		
Marital status;			0.723
Married	58 (93.5)	52 (94.5)	
Single	1 (1.6)	1 (1.8)	
Divorced	2 (3.2)	1 (1.8)	
Widowed	1 (1.6)	1 (1.8)	
Family history, n (%),			
Yes	21 (33.9)		
No	41 (66.1)		
Lesion localization; n (%),			
Left	23 (37.1)		
Right	27 (43.5)		
Bilaterally	12 (19.4)		
Morning stiffness, mean (SD), minute	27.19 ± 27.16		
Outcome, n (%),			
Cure	8 (12.9)		
Recurrence	54 (87.1)		

IGM, idiopathic granulomatous mastitis; HC, healthy controls; BMI, body mass index; *p* < 0.05.

**Table 3:** Comparison of laboratory characteristics between patients with IGM and healthy controls.

Variables, n (%)	IGM (n = 62)	HC (n = 55)	*p*-Value
WBC Count (/mm^3^)	7.71 ± 2.12	7.32 ± 1.79	0.290
Lymphocytes (/μL)	2.22 ± 0.63	2.25 ± 0.61	0.991
RBC count (/mm^3^)	4.76 ± 0.43	4.45 ± 0.23	0.001
Hemoglobin (g/dL)	11.97 ± 0.60	13.27 ± 1.30	0.001
Platelet count (10^3^/mm^3^)	297.37 ± 49.48	318.74 ± 42.54	0.014
ESR (mm/h)	19.27 ± 14.68	7.16 ± 3.01	0.001
CRP (mg/L)	9.91 ± 12.93	5.86 ± 3.70	0.027
Serum creatinine, mg/dL	0.66 ± 0.06	0.69 ± 0.02	0.001
e-GFR, mL/min/1.73 m2	110.45 ± 11.50	116.04 ± 20.45	0.076
RF (+) (>15 IU/mL)/Anti-CCP (+)	0	0	
C3 (mg/dL)	1.28 ± 0.20	1.30 ± 0.22	0.524
C4 (mg/dL)	26 ± 0.19	23 ± 0.07	0.437
ANA (-)	40 (64.5%)	50 (90.9%)	0.001
1/80 (+)	20 (32.3%)	5 (9.1%)	0.001
1/160 (+)	1 (1.6%)	0	0.348
1/320 (+)	1 (1.6%)	0	0.348
Anti-ds DNA (+)	0	0	0.064
Anti-Smith (+), suspicious	4 (6.5%)	0	0.055
Erytema nodosum	4 (6.5%)	0	0.055

IGM, idiopathic granulomatous mastitis; HC, healthy controls; WBC, white blood cells; RBC, red blood cells; ESR, erythrocyte sedimentation rate; CRP, C-reactive protein; e-GFR, estimated glomerular filtration rate; C3, complement 3; C4, complement 4; RF, rheumatoid factor; Anti-CCP, cyclic citrullinated peptide; ANA, antinuclear antibody; *p* < 0.05.

**Table 4:** SLERPI findings of patients with IGM and healthy controls.

Variables n (%)	IGM (n = 62)	HC (n = 55)	*p*
Malar rash or maculopapular rash	3 (4.8)	0	0.087
Subacute CLE or DLE	0	0	
Alopecia	21 (33.9)	10 (18.2%)	0.056
Mucosal ulcers	20 (32.3)	12 (21.8%)	0.209
Arthritis	37 (59.7)	1 (1.8%)	0.001
Serositis	1 (1.6)	0	0.348
Leucopenia < 4000/μL	1 (1.6)	0	0.290
Thrombocytopenia or autoimmune hemolytic anemia.	0	0	0.014
Neurological disorder	3 (4.8)	0	0.087
Proteinuria > 500 mg/24 h	0	0	0.044
ANA	22 (35.5)	5 (9.1%)	0.001
Low C3 and C4	(-) 0	(-) 0	0.417
Immunological disorder (any of anti-DNA, anti-Sm, anti-phospholipid antibodies)	9 (14.5)	1 (1.8%)	0.056
Interstitial lung disease	(-) 0	(-) 0	0.347

IGM, idiopathic granulomatous mastitis; HC, healthy controls; CLE, cutaneous lupus erythematosus; DLE, discoid lupus erythematosus; C3, complement 3; C4, complement 4; ANA, antinuclear antibody; *p* < 0.05.

**Table 5:** Comparison of SLERPI (+) and SLERPI (-) findings in patients with IGM.

Variables n (%)	SLERPI (+) ≥7 (n = 12)	SLERPI (-) <7 (n = 50)	*p*
Age, years, mean (SD)	33.33 ± 7.57	35.68 ± 8.52	0.386
BMI (kg/m^2^)	26.10 ± 2.79	27.40 ± 3.52	0.240
Malar rash or maculopapular rash	3	0	0.001
Subacute CLE or DLE	0	0	0.273
Alopecia	7 (58.3)	14 (28)	0.046
Mucosal ulcers	7 (58.3)	13 (26)	0.043
Arthritis	11 (91.7)	26 (52)	0.082
Serositis	1 (8.3)	0	0.040
Leucopenia < 4000/μL	0	0	0.637
Thrombocytopenia or autoimmune hemolytic anemia.	0	0	0.376
Neurological disorder	0	3 (6)	0.384
Proteinuria > 500 mg/24 h	0	0	0.519
ANA	10 (83.3)	12 (24)	0.001
Low C3 and C4	0	0	
Immunological disorder (any of the following: anti-DNA, anti-Sm, anti-phospholipid antibodies)	4 (33.3)	5 (10)	0.039
Interstitial lung disease	0	0	0.518

SLERPI, SLE risk probability index; BMI, body mass index; CLE, cutaneous lupus erythematosus; DLE, discoid lupus erythematosus; C3, complement 3; C4, complement 4; ANA, antinuclear antibody; *p* < 0.05.

## Data Availability

Data are available upon reasonable request.
